# The Influence of the Addition of Plant-Based Natural Fibers (Jute) on Biocemented Sand Using MICP Method

**DOI:** 10.3390/ma13184198

**Published:** 2020-09-21

**Authors:** Md Al Imran, Sivakumar Gowthaman, Kazunori Nakashima, Satoru Kawasaki

**Affiliations:** 1 Graduate School of Engineering, Hokkaido University, Sapporo 060-8628, Japan; gowtham1012@outlook.com; 2 Faculty of Engineering, Hokkaido University, Sapporo 060-8628, Japan; k.naka@eng.hokudai.ac.jp (K.N.); kawasaki@geo-er.eng.hokudai.ac.jp (S.K.)

**Keywords:** jute, MICP, ureolytic bacteria, biocement, natural plant fiber

## Abstract

The microbial-induced carbonate precipitation (MICP) method has gained intense attention in recent years as a safe and sustainable alternative for soil improvement and for use in construction materials. In this study, the effects of the addition of plant-based natural jute fibers to MICP-treated sand and the corresponding microstructures were measured to investigate their subsequent impacts on the MICP-treated biocemented sand. The fibers used were at 0%, 0.5%, 1.5%, 3%, 5%, 10%, and 20% by weight of the sand, while the fiber lengths were 5, 15, and 25 mm. The microbial interactions with the fibers, the CaCO_3_ precipitation trend, and the biocemented specimen (microstructure) were also evaluated based on the unconfined compressive strength (UCS) values, scanning electron microscopy (SEM), and fluorescence microscopy. The results of this study showed that the added jute fibers improved the engineering properties (ductility, toughness, and brittleness behavior) of the biocemented sand using MICP method. Furthermore, the fiber content more significantly affected the engineering properties of the MICP-treated sand than the fiber length. In this study, the optimal fiber content was 3%, whereas the optimal fiber length was s 15 mm. The SEM results indicated that the fiber facilitated the MICP process by bridging the pores in the calcareous sand, reduced the brittleness of the treated samples, and increased the mechanical properties of the biocemented sand. The results of this study could significantly contribute to further improvement of fiber-reinforced biocemented sand in geotechnical engineering field applications.

## 1. Introduction

Recently, significant interest in bio-mediated soil improvement has been highlighted as an innovative and effective approach for soil and ground improvement. Among the various bio-mediated soil development approaches, microbial-induced carbonate precipitation (MICP) has been recognized as a promising approach for soil improvement in recent years. The microbial urease hydrolyzes urea [CO(NH_2_)_2_] and produces ammonium and carbonate ions, and consequently increases the pH during the MICP, process resulting in an alkaline growing environment, which is favored for CaCO_3_ precipitation. The CaCO_3_ are the primary binding substances in between the sand particles, which lead to soil improvement.

Most of the previous studies [1,2,3,4] have focused on the impacts of various environmental factors on microorganism immobilization and strength improvement using several types of soil materials, including the capability of microorganisms to form CaCO_3_ within sand particles and pores; the relationship between the precipitated CaCO_3_ content and the strength of MICP-treated sand; the study of the engineering properties of MICP-treated sand, such as the volume, permeability, strength, and compressibility, which was assessed using introductory numerical simulations [5]. However, many recent experiments have demonstrated the mechanism of CaCO_3_ deposition and improvement of soil strength after curing samples using the MICP method. Earlier research also demonstrated the non-uniformity of precipitation of CaCO_3_ and brittle failure behavior of MICP-treated soil [6]. One study showed that the MICP-treated soil tended to fail at a low axial strain level in unconfined and triaxial compression tests. The axial stress of the samples also dropped rapidly after the peak stress, because most of the CaCO_3_ precipitated non-uniformly close to the influent of the specimen column and hindered the biocementation process in the deeper location of the specimen [7].

To improve this shortcoming, several studies have been conducted to improve the ductility and toughness of sand after MICP curing. Although sand solidified by microorganisms can reach a high strength, brittle failure of the substances mainly occurs and the strength is lost immediately, leading to safety hazards and problems. Therefore, the development of new mechanisms to improve the toughness, ductility, and durability of MICP-treated soil is a critical research need. The toughness, ductility, and durability of MICP-treated soil can be improved by the addition of fibrous materials. Earlier studies showed that using fibrous materials increased bacterial interactions with the cementation solution and facilitated uniform CaCO_3_ precipitation within the sand pores in MICP-treated soil [8]. Several studies have also revealed that the addition of fiber materials can substantially increase the engineering properties of MICP-treated soil (for example the shear strength, ductility, brittleness behavior, internal friction, and rigidity) [9]. However, to address these challenges, most of these previous studies focused on using synthetic-polymer-based fiber materials, such as non-woven geotextile fiber, steel fiber, polypropylene fiber, glass fiber, and carbon fiber, which are associated with relatively high costs, health concerns, and environmental hazards [10,11]. Moreover, to date, only a few experiments have concentrated on the improvement of the engineering properties of soils treated with the MICP method using fiber materials. Therefore, developing a new approach and identifying cheap, readily available fiber while considering safety, environmental considerations, and sustainability will require further investigations. A detailed analysis to improve the drawbacks of MICP-treated sand via the inclusion of fibers was inspired by the current deficiencies in the traditional MICP process.

This study considered the use of natural jute fiber (*Corchorus capsularis*), because jute fiber has excellent physical mechanical properties and high resistance capacity (temperature, pH, salinity) and is readily available, inexpensive, light weight, sustainable and eco-friendly, requiring low amounts of energy [12,13].

The primary objectives of this work were to investigate the effects of a natural fiber (jute) on the MICP-treated soil. To improve the engineering properties (ductility, toughness, and brittleness behavior) of the biocemented sand specimen in terms of the fiber length and content (ratio), carbonate precipitation patterns and interactions within the microorganisms were investigated in this study. The mechanical properties of the MICP-treated sand, the microstructure of the specimen, and the interactions between the fiber and microorganisms were investigated and analyzed using unconfined compressive strength (UCS), scanning electron microscopy (SEM), and fluorescence microscopy.

## 2. Materials and Methods

### 2.1. Fiber

**Figure 1:**
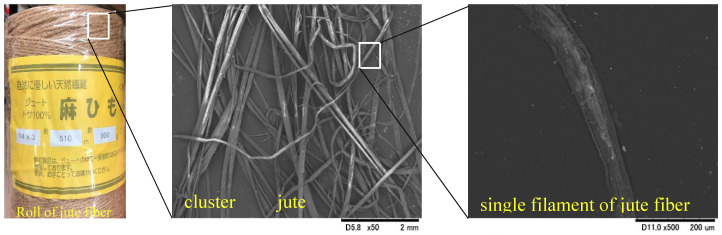
Appearance and microstructure images of jute fibers, obtained using SEM.

Previous research studies have indicated that jute fibers of appropriate length and content can substantially increase a soil’s engineering properties, and adding fibrous material can contribute to a significant reduction in construction costs [14,15] by improving the engineering properties of treated samples. Moreover, jute fibers have a high initial modulus, high consistency in terms of tenacity and tensile strength, high rigidity, and a lower percentage of elongation during breakage, leading to their wide use in soil improvement. The properties of jube fibers, as well as their availability, cost, and environmental friendliness, were the motivation for using jute fibers in this study. Locally available jute fibers (100% natural) were used in this study (Figure 1) without any chemical treatment. The jute fibers were purchased from DCM Homac Co., Ltd., Sapporo, Japan, and the fibers were collected by Hayase Industries, Ltd., Tsuyama, Japan. The microstructure of the jute fibers was examined through scanning electron microscope images. SEM images of clusters and single filaments of jute fibers are presented in Figure 1.

**Figure 2:**
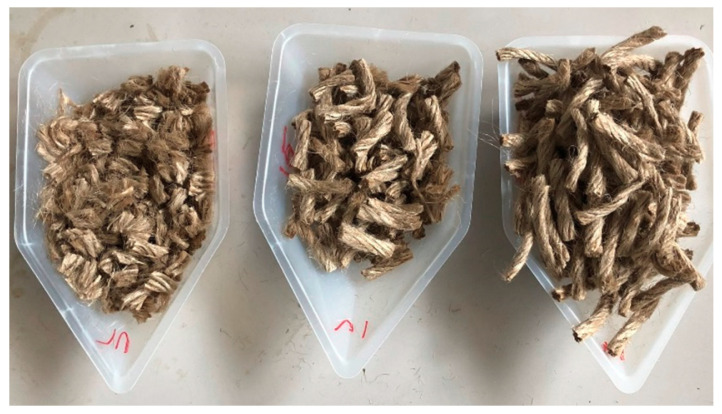
The physical appearance of the jute fibers used in this study, which were cut into different length.

The key features of the jute fibers used in this study are shown in Table 1. It was shown that the efficiency of the fibers used to improve the soil highly depends on the properties of the fibers, such as the nature of fibers, the fiber length, and the fiber ratio [16]. The jute fibers were cut into three different lengths (5, 15, and 25 mm) and several percentages (content) by weight (0.5%, 1.5%, 3%, 5%, 10% and 20%) were used for mixture with “Mikawa” sand particles. The physical appearance of the prepared jute fiber samples is shown in Figure 2. All the jute fibers used in this study were placed in a dryer at 60 °C for 24 h before being mixed with the sand and receiving MICP treatment.

### 2.2. Microorganisms and Soil Properties

**Figure 3:**
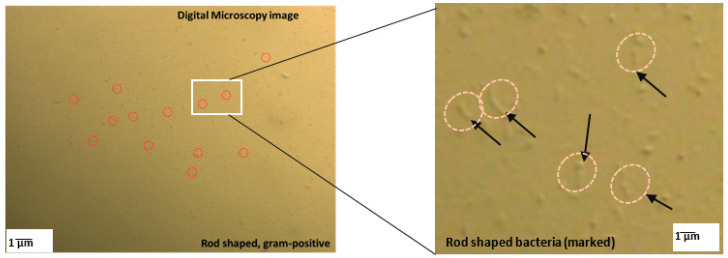
Digital microscopic image of the microorganism used in this study.

The bacteria used in this study was *Micrococcus yunnanensis* (hereafter denoted as G1), which was isolated from the coastal area of Porto Rafti, Greece [17]. The key features of this bacteria (Figure 3) are that it is known to exhibit comparatively high urease activity with salt-tolerant properties and can survive for extended periods of time in various temperature and pH conditions, as well as in nutrient-deficient conditions [18]. Liquid ZoBell2216 medium was used as the culture solution for the selected bacterial species. The culture medium was dissolved with hi-polypeptone (5.0 g/L), FePO_4_ (0.1 g/L), and yeast extract (1.0 g/L). The components were mixed with artificial seawater and the pH was maintained at 7.6–7.8. The bacterial cells were precultured (using the ZoBell2216 medium) for 24 h at 30 °C in a shaker at 160 rpm. The precultured bacterial cells were then transferred (1 mL) into 100 mL of fresh ZoBell2216 medium and incubated at 30 °C at 160 rpm. The prepared bacterial culture solution was used for the MICP process. During the cultivation, the bacterial cell growth (OD_600_) was determined and adjusted (by approximately 6) using a UV-Vis spectrophotometer (V-730, JASCO Corporation, Tokyo, Japan) and urease activity (1.5 ± 3 U/mL) was measured using the indophenol method [19].

**Figure 4:**
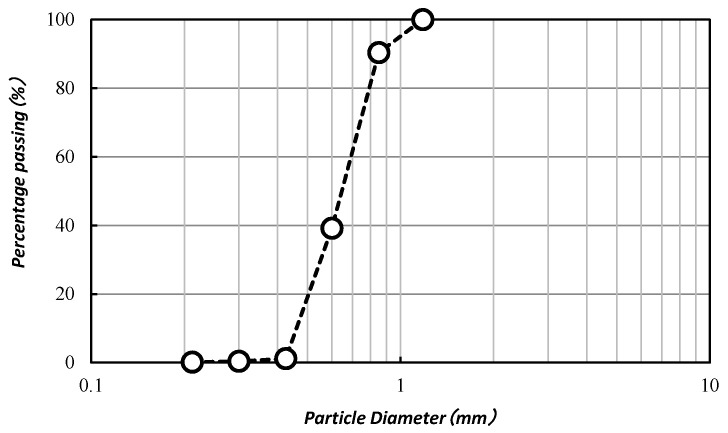
The grain size distribution of “Mikawa” sand.

The soil used in this study was commercially available “Mikawa” sand. The maximum and minimum dry densities of the sand were 1.476 and 1.256 g/cm^3^. The particle density and mean diameter were 2.66 g/cm^3^ and 870 μm, respectively. The grain size distribution of “Mikawa” sand is presented in Figure 4. Before the MICP process, the sand was dried in an oven dryer at 110 °C for 24 h.

### 2.3. Effects of Jute Fibers on the Process of CaCO_3_ Precipitation

To investigate the interactions between jute fibers and CaCO_3_, equal concentrations (0.5 mol/L) of CaCl_2_ and urea solutions were used for the precipitation test in test tubes with and without fiber. The total volume of the mixers was adjusted up to 10 mL using distilled water, samples were kept in the shaker for 48 h at 30 °C, and the rpm was kept at 160. The testing conditions are shown in Table 2 and Table 3, showing the fiber content and length, respectively. After 48 h, the resulting mixture was centrifuged to collect the crystal precipitate and the supernatant of solutions from the tube was removed separately using filter paper (Whatman filter paper, 11 μm (Global Life Sciences Technologies Ltd., Tokyo, Japan)). Both the filter papers and the tubes with the precipitate were oven-dried for 24 h at 110 °C, then subsequently the dry weights of the crystals were measured. The weight of the precipitated crystal was determined by contrasting the empty weight of the tube from the dry weight of the tube and the filter paper’s dry weight. Using scanning electron microscopy (SEM; MiniscopeTM3000, Hitachi, Tokyo, Japan), the morphologies of crystals and jute fibers were analyzed. All the experiments were done in triplicate, and the mean value was plotted accordingly. Standard deviation was used to represent the error bars.

### 2.4. Sample Preparation

**Figure 5:**
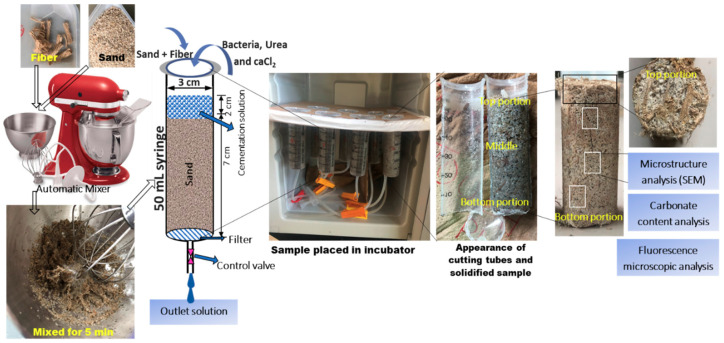
Sample preparation and test setup for the microbial-induced carbonate precipitation (MICP) process with the addition of jute fibers.

The designed materials and test setup for the MICP treatment are shown in Figure 5. The dried “Mikawa” sand (75 ± 5 g) was taken into a 50 mL standard syringe tube (diameter 3 cm, height 10 cm). In each case, oven-dried samples (as described earlier) were compacted into 3 layers by applying a hammer shock on each layer of the sand. A lab-grade filter paper was used to cover the bottom portion of each column. Each sand column was filled with consistently mixed jute fibers using an automatic mixer (kitchen aid 9KSM160 series), with different fiber lengths and contents used. To neutralize the electrostatic charge of fibers and sand grains, 10 mL of (DW) de-ionized water was added during the mixing process to ensure uniform distribution of fibers within the soil matrix.

Thereafter, 12 mL of bacterial culture solution (ZoBEll2216E) was injected from the top of the syringe and superfluous solutions were drained out at a controlled rate to achieve bacterial stabilization (approximately 2 h) within the soil matrix. At the later injection phase, 16 mL of cementation solution (30.0 g/L of urea, 55.0 g/L of CaCl_2_, and 3.0 g/L of Bacto nutrient broth) was injected into the samples. The injected solution was kept at approximately 2 mL above the surface of the sand and maintained in a fully saturated condition. The prepared samples were kept in an incubator at 30 °C for 14 days.

The cementation solution was injected and drained every day for 14 days continuously. The pH values and Ca^2+^ concentrations from the outlet were measured every day. After 14 days of curing, the UCS of the samples were measured using an automated Instron 2511-308 load cell (Norwood, MA, USA) following the ASTM D7012 2014 standard. The axial strain rate was 0.036 mm/min until reaching the failure condition (critical stage). The testing conditions are presented in Table 4 and Table 5, showing the fiber content and length, respectively.

The bacterial retention capacity (bacterial immobilization) during the MICP treatment was measured by the difference between the primary injected bacterial solution (OD_600_) and the effluent solution (OD_600_). To understand the cementation behavior of the treated samples, a pair of 0.5 MHz transducers with oscilloscope were also used to measure the primary and secondary shear wave velocities (*V*p, *V*s) of the treated samples. The transmitted signal was a 200 kHz square wave across the length of the cylindrical specimen (~6 cm). The velocities (*V*p, *V*s) of the treated specimens were calculated using SonicViewer-SX:5251 by measuring the time differences. The improvement ratio (IR) was calculated using the difference between the comparative values of the treated samples and the comparative values of the untreated samples. All the measurements were conducted after the samples were removed from the syringes and in dried condition.

The morphology, microstructure, and interactions of the precipitated CaCO_3_, sand particles, and jute fibers were investigated using scanning electron microscopy (SEM). All the experiments were done in triplicate, and the mean value was plotted accordingly. The standard deviation was used to represent the error bars. The behavior of microorganisms and the effects of adding fibers to the bacterial culture solution were also investigated using an automatic fluorescence microscope (BZ-X800, KEYENCE Corporation, Osaka, Japan).

**Figure 6:**
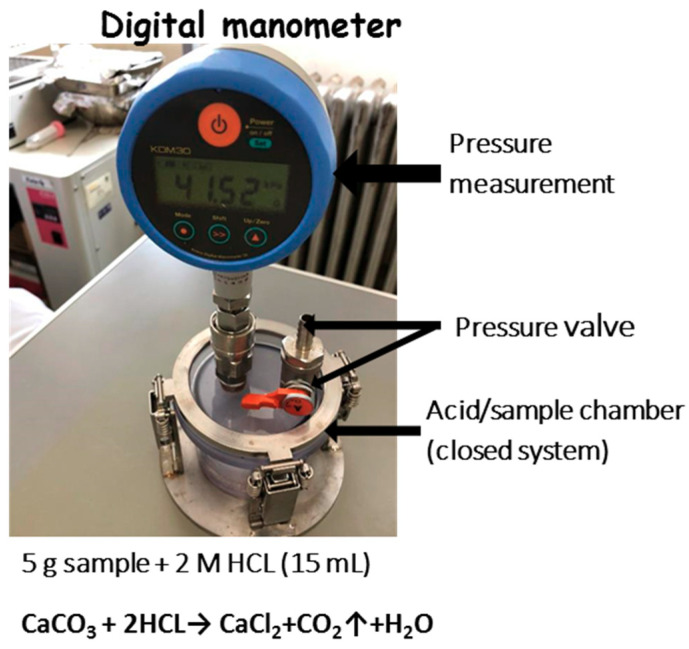
Digital manometer setup used to measure the CaCO_3_ content.

The precipitated CaCO_3_ content of the MICP-treated specimens were measured using a simplified digital manometer device (Figure 6) under constant volume and temperature, followed by the ASTM standard method (ASTM D4373-14) [20].

## 3. Results and Discussion

### 3.1. Effects of Jute Fibers on the Process of CaCO_3_ Precipitation

**Figure 7:**
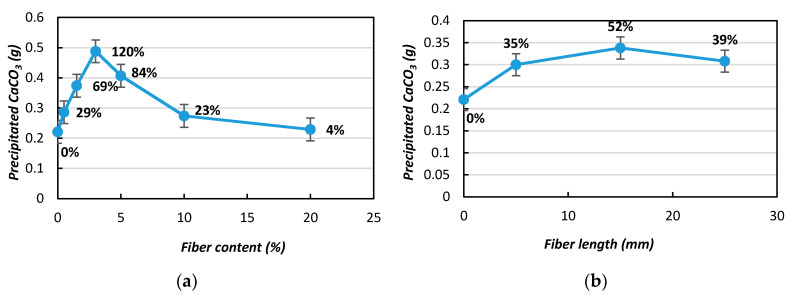
Effects of fiber content on CaCO_3_ precipitation with the addition of jute fibers: (**a**) fiber content; (**b**) fiber length.

Studies have shown that the trend of CaCO_3_ precipitation during the MICP process is greatly influenced by the fiber length and content (%) [21]. Therefore, it is essential to investigate the trends in CaCO_3_ precipitation for individual bacterial species, because the CaCO_3_ acts as the main binding material in between the substrate particles during the MICP process, leading to soil improvement. The effects of using the same fiber length and different fiber contents on CaCO_3_ precipitation are presented in Figure 7. As can be shown, the amounts of precipitated CaCO_3_ crystals varied significantly depending on the fiber content. By increasing the fiber content by 0.5%, the CaCO_3_ precipitation was increased by approximately 29%, and reached 120% with the addition of 3% jute fibers compared to without fiber addition (Figure 7a). However, further addition of jute fibers decreased the amount of CaCO_3_ (5% and 10% addition resulted in 84% and 23% improvements, respectively). With the addition of 20% jute fiber, the precipitation content dropped by 4%.

From this study, it was observed that the effect of CaCO_3_ precipitation with 3% fiber is the best condition. From this study, it was also revealed that the higher fiber content intensely influenced the CaCO_3_ precipitation. This study suggested that the optimum CaCO_3_ content was obtained with an increase in fiber content up to 3%, while further increases of the fiber content led to decreased CaCO_3_ precipitation compared to the other fiber content conditions. The reason for this phenomenon is that natural jute fibers have a non-regular cross sectional geometry and contain some chemical compounds such as pectin that act as accelerators (pectin breaks down into sugar and is used as the nutrient for the bacteria) [22] and inhibitors (excess amounts of sugar and other chemical compounds released from the jute fiber) of the microorganism growth (depending on the fiber content), which is also supported by previous studies [23].

Figure 7b shows the amounts of CaCO_3_ precipitation with the same fiber content but variations in length (5, 15, and 25 mm, respectively). Figure 7b shows that the CaCO_3_ precipitation increased to 35%, 52%, and 39% with 3% fiber content and lengths of 5, 15, and 25 mm, respectively, compared to without addition. The variation in the CaCO_3_ precipitation amounts within the same set of samples was lower than when the fiber content was varied. The findings of this study indicate that the content (weight %) of the fiber addition is more important than the fiber length in the MICP treatment in order to promote the precipitation of CaCO_3_, which is in good agreement with previous studies [24,25].

**Figure 8:**
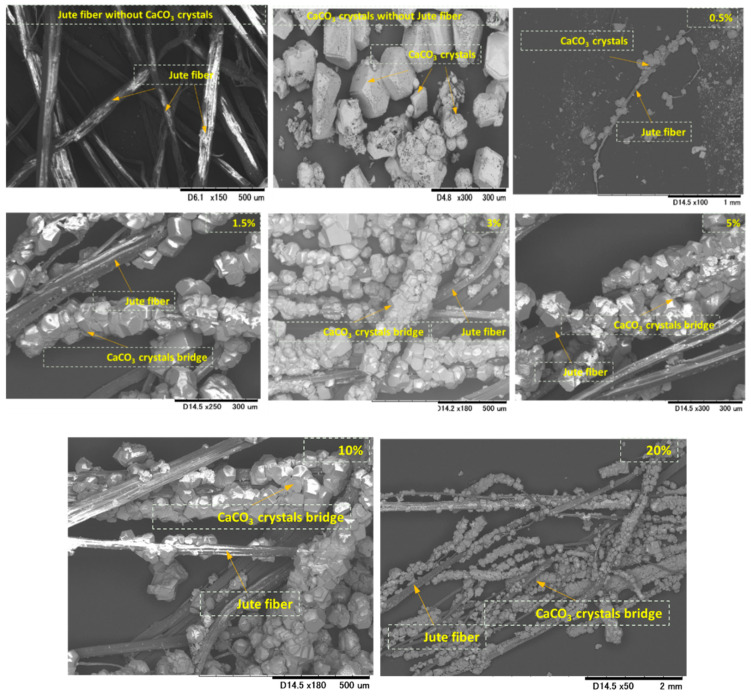
SEM images of precipitated CaCO_3_ with and without jute fibers after the MICP process (without sand materials).

The microstructure analysis and interactions of precipitated CaCO_3_ with jute fibers are shown in Figure 8 (with fiber and without fiber). The test results indicate that the precipitated crystals are irregular in shape and size and are coated around the jute fibers. The precipitated CaCO_3_ also formed a CaCO_3_ bridge, which could be very effective for binding and filling the void space in between the sand particles. Similar observations were also reported in previous studies [11,26]. The adsorption capacity of the microorganisms and CaCO_3_ precipitation to the fibers was greatly influenced by the surface microstructures of the fiber. Different fiber and surface microstructures lead to different CaCO_3_ precipitation patterns [27]. In addition, the reduction in the void space due to the CaCO_3_ precipitation could be considered to be a primary strengthening factor [28,29] in the reduced brittleness behavior of the MICP-treated sand.

### 3.2. Effects of Fiber Inclusions on Microorganisms

**Figure 9:**
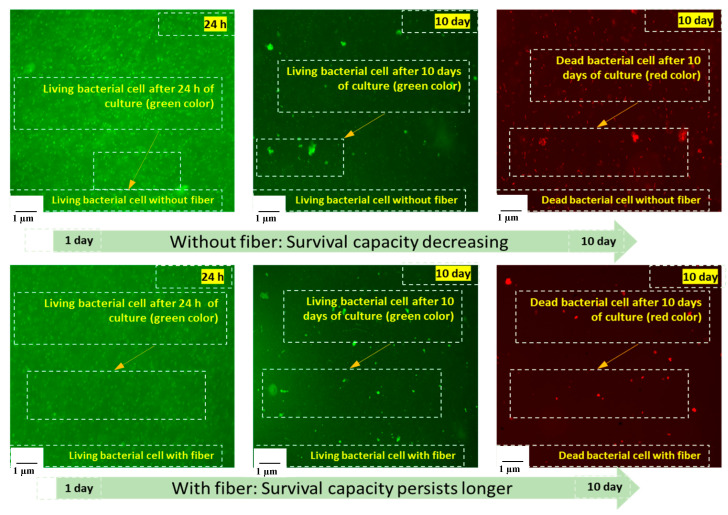
Fluorescence microscopic images living and dead bacterial cell (with and without fiber).

The fluorescence microscopic images of the cultured bacterial cells (with and without fibers) are shown in Figure 9. The images show that the bacterial survival capacity persists for longer with the addition of jute fibers. The number of dead bacterial cells was also reduced after 10 days of cultivation with the addition of the fibers. The action behind this characteristic is the availability of biopolymers (cellulose, hemicellulose, lignin, pectin, and waxy substances) in the jute fibers [30]. When jute fibers are steeped in water, the water-soluble carbohydrate compounds (D-glucosidic bonds and hydroxyl groups) and biopolymers are broken down into simple sugars (galactose) by a bio-chemical mechanism [31], acting as a source of nutrient for the bacteria, which is essential for the bacteria to survive for a longer time than usual. However, a further multidisciplinary assessment could be conducted to quantify this mechanism.

### 3.3. Variations in Strength after Fiber Inclusion in the MICP-Treated Sample

**Figure 10:**
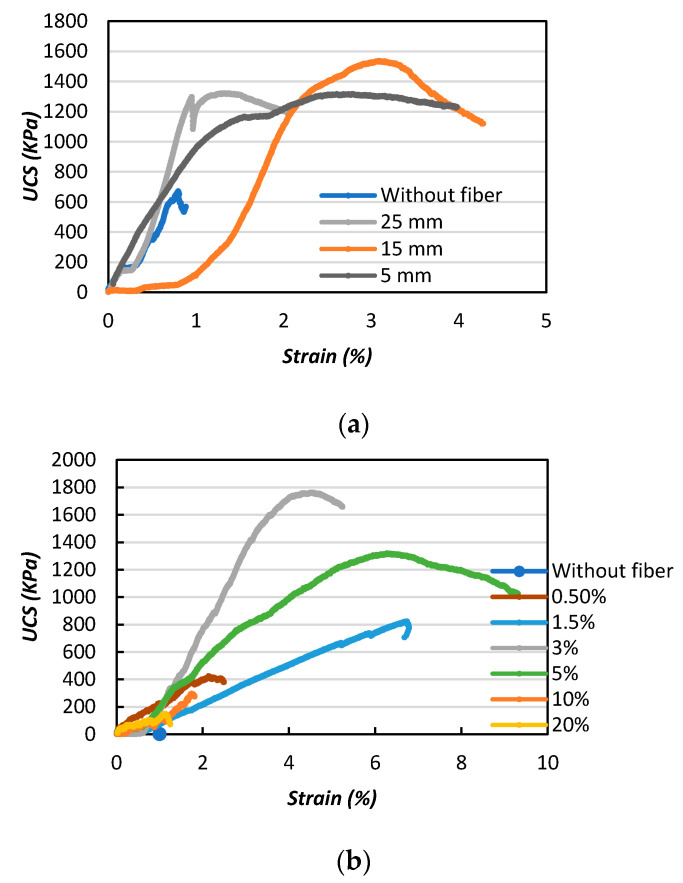
Effects of fiber on the unconfined compressive strength (UCS) of the biocemented sand with the addition of jute fibers: (**a**) fiber length; (**b**) fiber content.

The stress–strain curves of the MICP-treated samples with different fiber lengths and contents are presented in Figure 10, which shows that the MICP-treated samples were significantly influenced by the addition of jute fibers (depending on the fiber content and length). The stress–strain curve of the MICP-treated biocemented sand without fiber was gradually compacted with increasing strain and stress, then failure occurred, which was considered as typical brittle failure. However, by increasing the fiber length (5, 15, and 25 mm), the stress on the biocemented sand reached the maximum strength and then entered the residual deformation stage. Failure occurred more slowly compared to the samples without fibers (Figure 10a). The slower rate of failure indicated improvement of the ductility behavior of the samples. In addition, as shown in Figure 10a, by increasing the fiber length, the strength (UCS) declined. The results of this study indicated that the addition of long fibers to the MICP-treated samples could meant they could be easily bent and the fibers eventually clustered within the sand, having a negative impact on the MICP treatment and leading to declines in the UCS. The results of this study were significant compared to a previous study [32].

Figure 10b shows that the unconfined compressive strength (UCS) of the biocemented sample initially increased (from 0.5% fiber content) and reached its maximum strength (UCS) with a fiber content of 3%. The unconfined compressive strength (UCS) interestingly decreased with further addition to the fiber content (up to a fiber content of 20%).

The reason for this was that the fiber was randomly distributed within the sand matrix by the interleaving mechanism and due to the cross-sectional geometry [33]. This mechanism led to several interlacing points forming between fibers, then a spatial distribution network and spatial stress area formed that were able to increase the bacterial retention and survival capacity (as mentioned earlier), meaning the sample was able to hold more bacteria (which resulted in increased CaCO_3_) than samples without fibers. The stress area and the network controlled the deformation of the sand and increased the ductile behavior of the MICP-treated biocemented sand.

**Figure 11:**
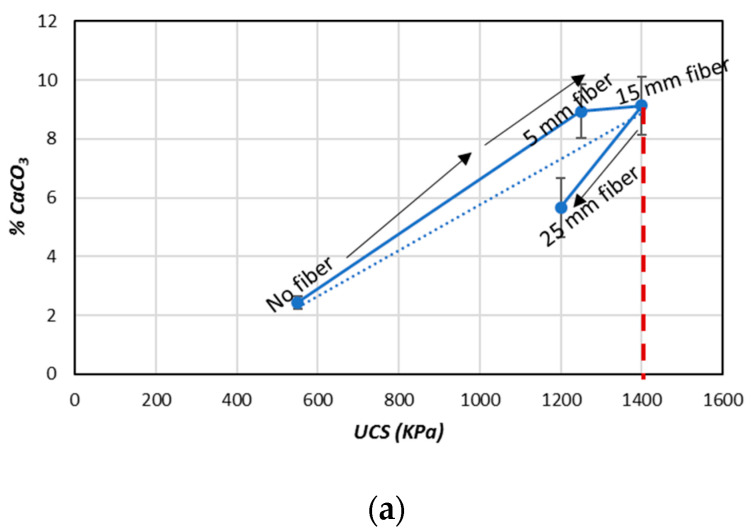

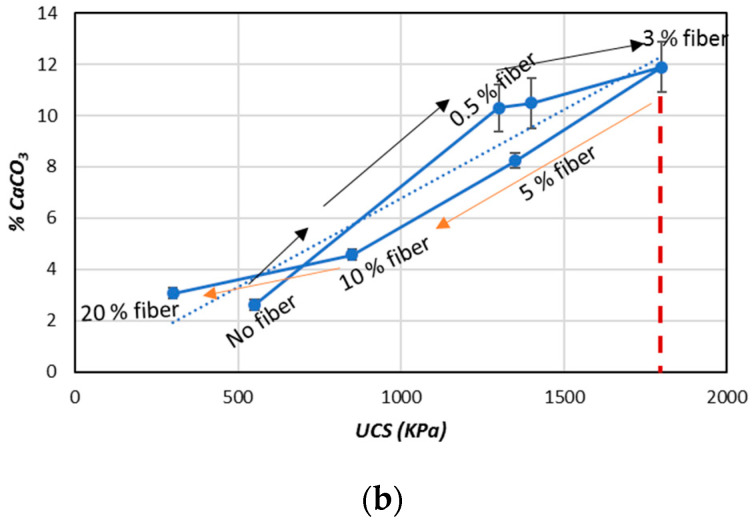
Average (%) CaCO_3_ values for the biocemented sand sample after MICP treatment with the addition of jute fibers: (**a**) fiber length; (**b**) fiber content.

The relationship between the estimated UCS and the CaCO_3_ (%) from the results obtained in this study (considering the fiber length and content) is shown in Figure 11. By increasing the fiber length (5–15 mm), the CaCO_3_ (%) increased and the UCS also increased. The maximum UCS was observed with a 15 mm fiber length (Figure 11a). By further increasing the fiber length (25 mm), the CaCO_3_ (%) decreased and the UCS was dropped. In Figure 11b, it was also shown that the fiber content played a considerable role in the CaCO_3_ (%) precipitation process and also improved the UCS. With the increases of the fiber content (0.5–3%), the CaCO_3_ content also increased. As a result of the increasing CaCO_3_, the UCS values of the treated samples also increased until reaching the maximum point (approximately 1.6 MPa). Further increases of the fiber content (5–20%) caused the CaCO_3_ (%) precipitation and UCS of the treated samples to decrease [33,34]. The findings of this study clearly reveal that the fiber content (% weight) played a more significant role compared to the fiber length in terms of influencing the amount of CaCO_3_ precipitation and improving the UCS in the treated samples.

**Figure 12:**
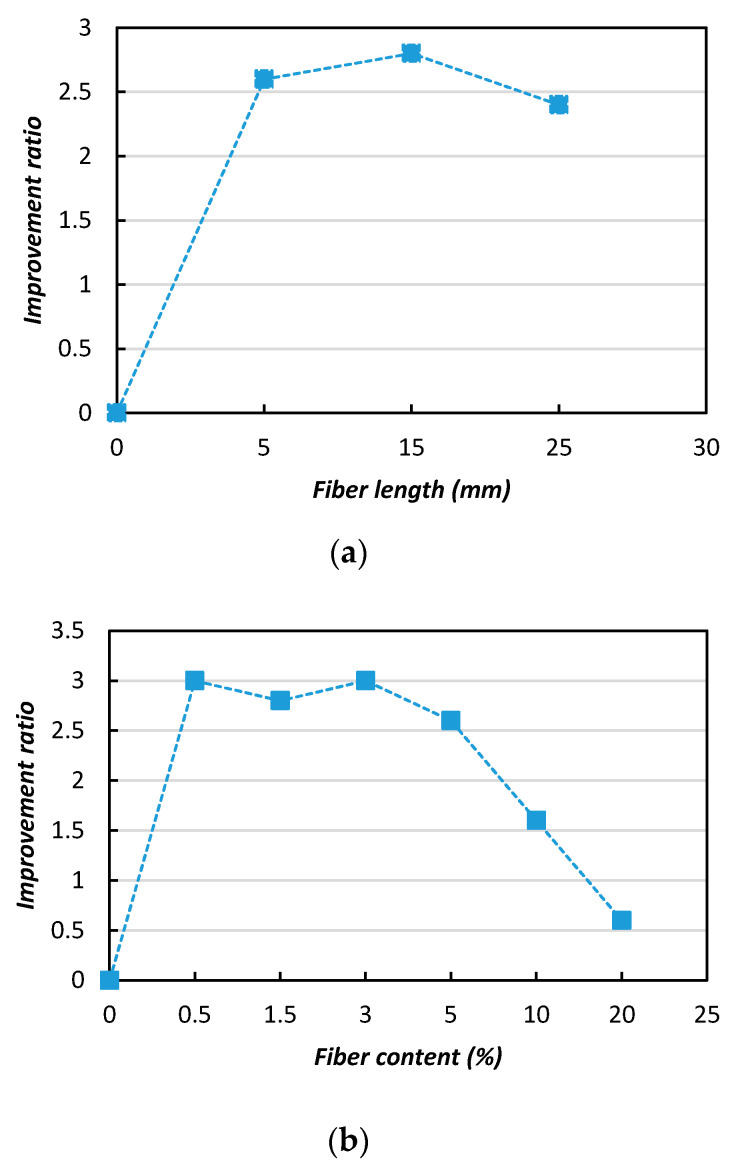
Improvement ratios (IRs) of the MICP-treated biocemented sample strength (UCS) with the addition of jute fibers: (**a**) fiber length; (**b**) fiber content.

Figure 12 shows the effects of the fiber length on the soil’s strength improvement (UCS). In the Figure 12a, it can be observed that the improvement ratio (IR) increased from 2.5 to 3 with appropriate increases of the fiber length (5–15 mm), however with further length increases the IR value dropped and a negative influence was evident. In Figure 12b, it can be observed that the IR value was indicates the significant influence of the fiber content in the improvement of the soil strength. A relatively greater improvements were achieved with the 0.5 and 3% additions of natural jute fibers, with improvement ratios of 2.5 and 3.1, respectively.

**Figure 13:**
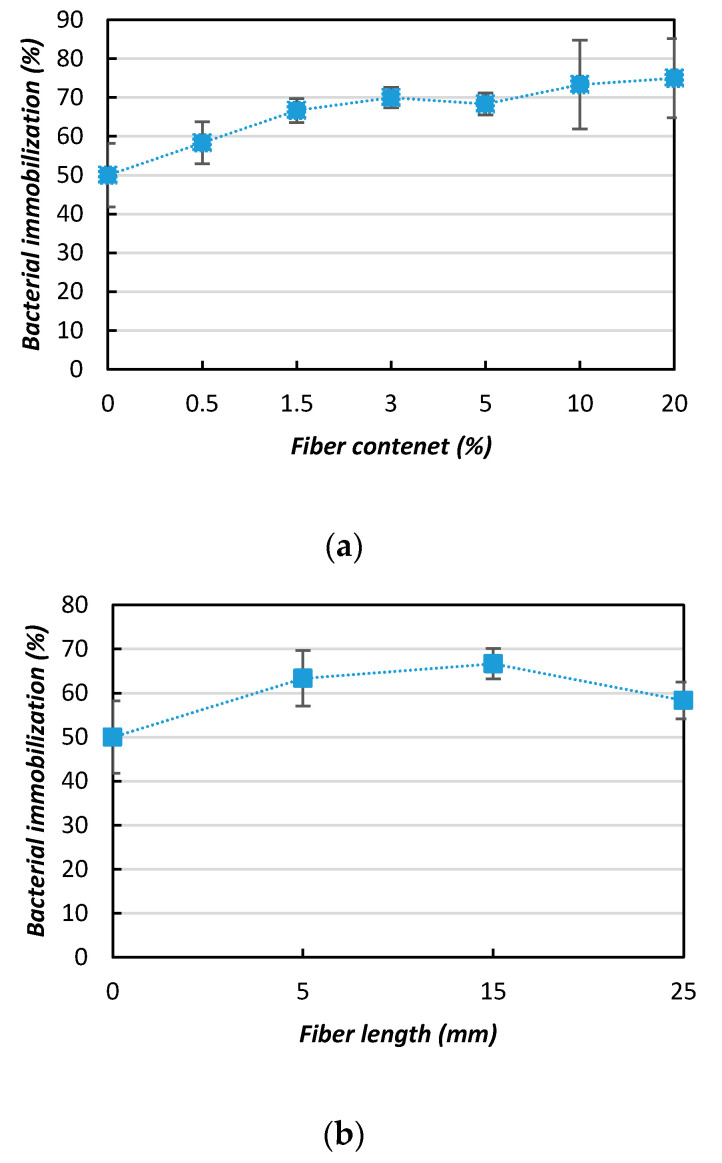
Influence of bacterial retention capacity with the addition of jute fibers: (**a**) fiber content; (**b**) fiber length.

In general, the results of this study suggest that the mixing of natural jute fibers with the MICP method could significantly improve biocemented sand, provided the appropriate length (i.e., 15 mm) and content (i.e., 3% by weight of sand) are used. The reason is that when jute fibers are mixed with sand, the cohesion, friction, and interface between the jute fibers and sand particles increase (depending on the fiber length and content). As a result, the frequent bacterial movement ensures and enhances the immobilization of the bacteria within the soil matrix (Figure 13a). The increased bacterial immobilization accelerates the uniform distribution of CaCO_3_ within the sand matrix and consequently increases the effectiveness of the soil (in terms of the UCS, ductility, etc.). However, if the fiber content is too high (i.e., 20%), the bacterial movement is hindered, resulting in uneven distribution of the bacteria within the soil matrix. Similarly, the CaCO_3_ precipitation occurs non-uniformly. In addition, increasing the fiber length by too much (i.e., 25 mm) reduces the efficiency of bacterial immobilization (Figure 13b) because of the uneven distribution of the fibers and the decreased retention capacity of the bacteria. As a result, the effectiveness of the soil’s engineering properties is also decreased [34].

### 3.4. Effects of Bacterial Immobilization on CaCO_3_ Precipitation

**Figure 14:**
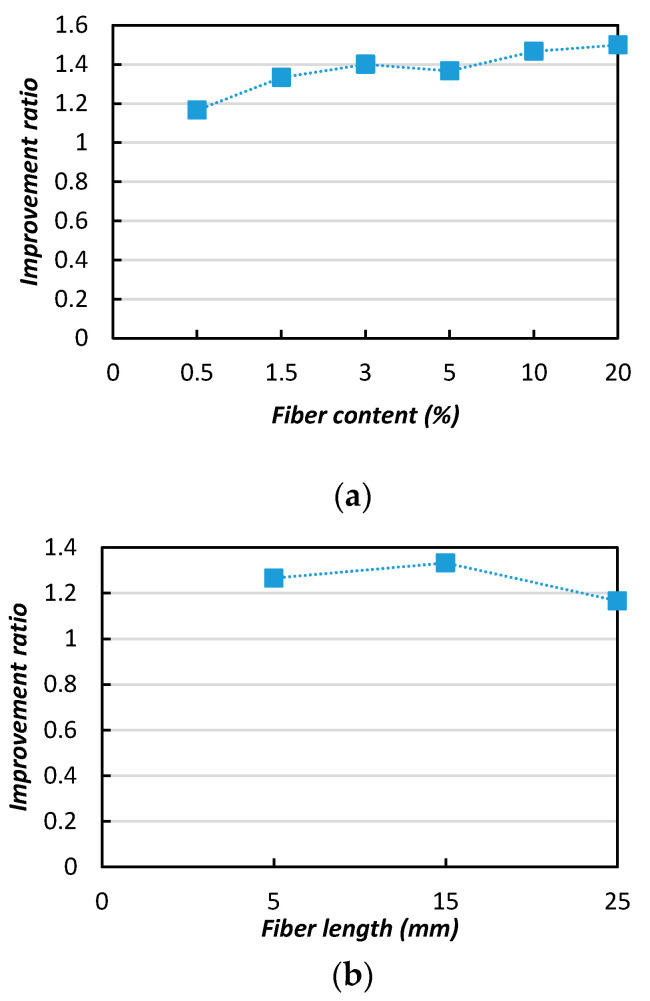
Improvement ratios for bacterial immobilization with the addition of jute fibers: (**a**) fiber content; (**b**) fiber length.

Figure 14 summarizes the variations in the bacterial immobilization improvement ratios (in terms of retention capacity) with respect to different fiber inclusions. Figure 14 shows that for pure the MICP sample (without fiber addition), approximately 50% of the bacteria were flushed out and the bacterial retention capacity (immobilization) increased with the fiber content (Figure 14a), yielding average improvement ratio values of 1.2 to 1.6 (with fiber contents ranging from 0.5 to 20%). Moreover, increasing the fiber length (from 5 to 25 mm) decreased the bacterial immobilization (retention capacity) (Figure 14b), giving an improvement ratio of 1.2 with the 15 mm fiber length. This study showed that the bacterial immobilization (retention capacity) was increased in the presence of fibers, as was also reported in previous studies [35,36].

**Figure 15:**
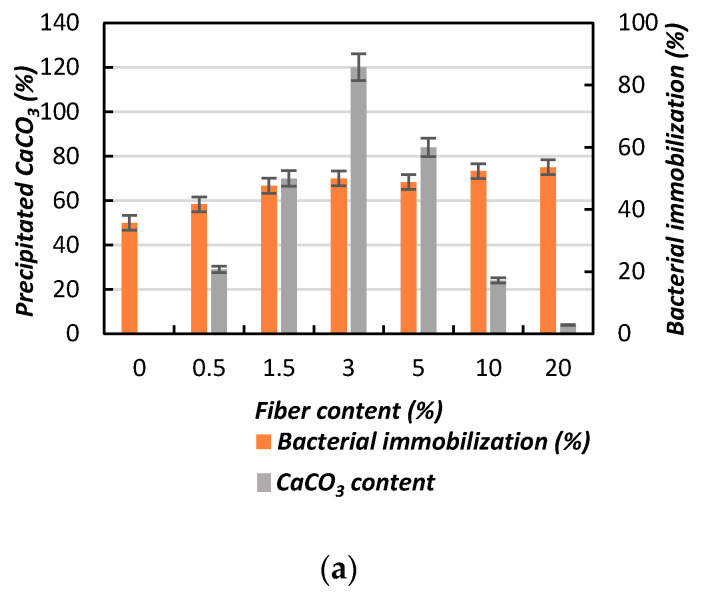

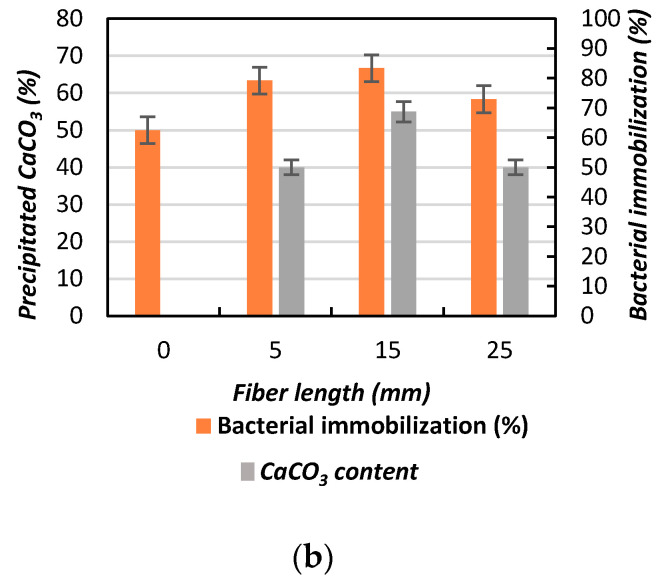
Effect of bacterial immobilization on the CaCO_3_ content with the addition of jute fibers: (**a**) fiber content; (**b**) fiber length.

Figure 15 shows the effect of bacterial immobilization on the CaCO_3_ content with the addition of jute fibers, considering both the fiber content (Figure 15a) and fiber length (Figure 15b). Figure 15a shows that the CaCO_3_ content increased with increasing fiber content up to a certain amount. In this study, 3% fiber content result in the maximum amount of CaCO_3_ (Figure 15a). With further increases of the fiber content, the bacterial movement [31] became stuck (due to the fiber structure, as described earlier), resulting in decreased CaCO_3_ content within the soil matrix. A similar process was also observed when increasing the fiber length (Figure 15b).

In this study, it was demonstrated that the production of CaCO_3_ was greatly influenced by the bacterial immobilization capacity (retention ability), and the appropriate fiber content and length resulted in an increased CaCO_3_ content.

### 3.5. Microstructure Analysis

**Figure 16:**
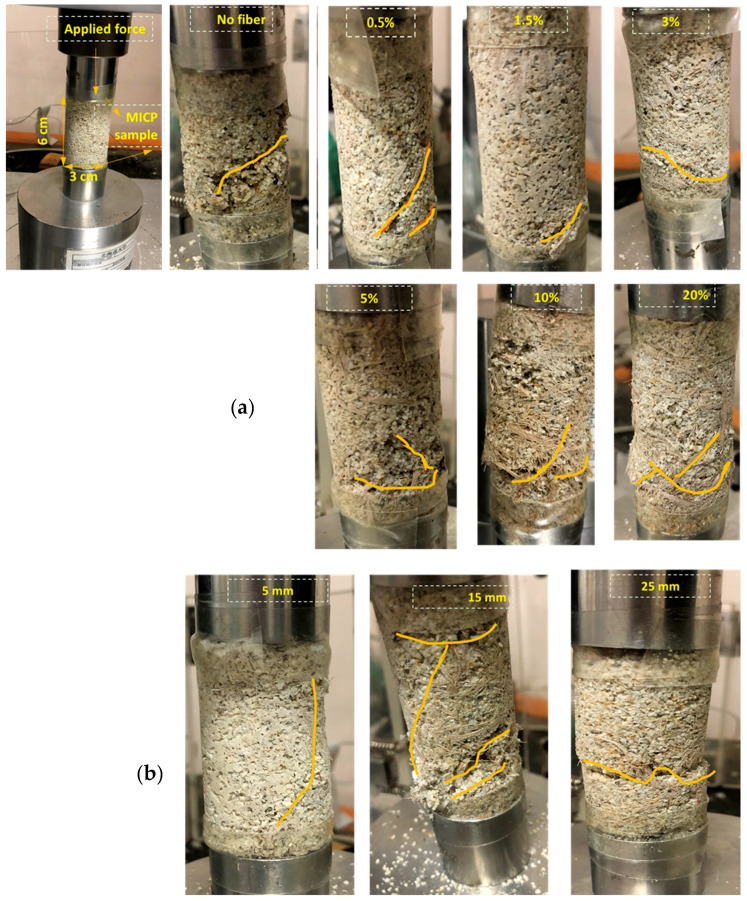
Failure behavior of the MICP-treated sample with addition of jute fibers: (**a**) fiber content; (**b**) fiber length.

The fracture morphologies of the biocemented samples with different fiber contents (0, 0.5, 1.5, 3, 5, 10, and 20%) and lengths (5, 15, and 25 mm) are also compared in Figure 16a,b, which shows the results of the unconfined compressive strength test. All the experiments were conducted in triplicate following the ASTM D2166 standard method.

In Figure 16 (considering both the fiber content and length), it can be seen that the addition of jute fibers in the MICP-treated sample significantly improved the unconfined compressive strength, and because of the lower biocementation level, the fractures generally started from the lower end (Figure 16a). Without the fibers, the fractures appeared throughout the whole sample from the bottom upward, suggested brittle failure of the samples. The results also showed that the fracture morphologies of the biocemented samples were closely interlinked with the different fiber contents (Figure 16a) and lengths (Figure 16b), due to the interaction and friction within the sand–fiber matrix. With increasing fiber content (i.e., 0.5–5%), the fiber formed a three-dimensional (3D) grid within the soil matrix, which restricted the development of the failure pattern and effectively improved the strength of the soil, while enhancing the brittleness delayed the overall damages of the MICP-treated sample [32]. However, further increasing the fiber content (i.e., 10–20%) resulted in ‘‘bulging’’ behavior. Regarding the fiber length, the use of short fibers (i.e, 5–15 mm) resulted in significant improvements for the biocemented specimen. The use of long fibers (i.e., 25 mm) led to the sudden fracture of the sample due to uneven distribution and bundles becoming entangled during the sample preparation. Therefore, for the actual engineering application, it is important to determine the optimum fiber content and length to be added to the soil in order to obtain the maximum effect.

**Figure 17:**
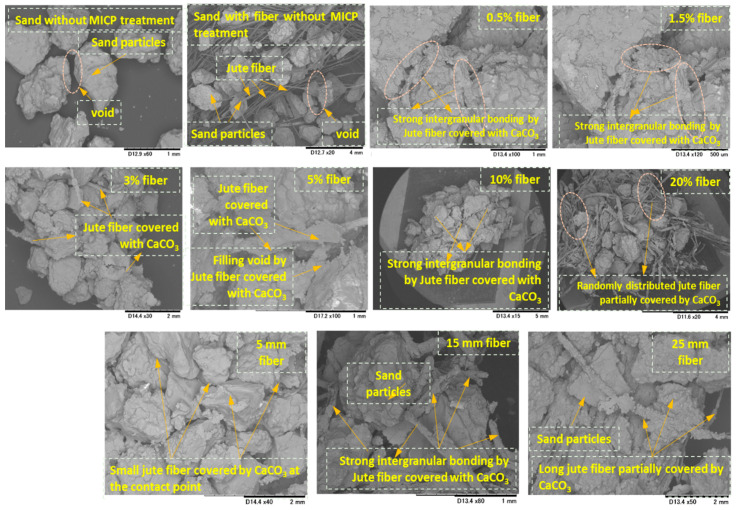
SEM images of MICP-treated biocemented samples with the addition of jute fibers (considering different lengths and contents) and distribution of CaCO_3_ within the sand matrix.

Figure 17 shows the SEM images of MICP-treated biocemented samples with the addition of jute fibers (considering the length and content) and the distribution of CaCO_3_ within the sand matrix. The images also show sand particles without MICP treatment, sand particles with fiber without MICP treatment, and biocemented sand particles with different fiber contents and lengths. From the microscopic images, it can be seen that the void spaces were dominant in both sand–fiber matrixes without MICP treatment. After the MICP treatment, the fibers were covered by CaCO_3_ crystals (similar to a bridge). The CaCO_3_ crystal bridge provided strong bonding between the sand particles and also filled the void space. Consequently, the cementation level of the treated sample increased significantly.

**Figure 18:**
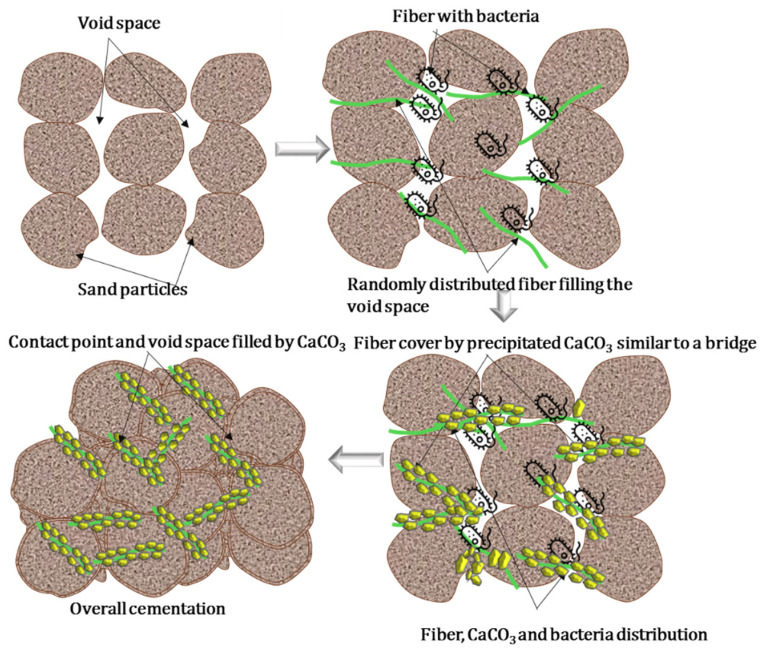
Schematic diagram of biocemented sample with the addition of jute fibers.

The overall interactions and improvement of the MICP-treated sand are presented more clearly by a schematic diagram in Figure 18. By increasing the fiber content (up to a certain amount) and fiber length (up to a certain length), more CaCO_3_ precipitation occurred in between the soil pore spaces and contact points, resulting in the soil having enhanced engineering properties. The results are also validated by the primary and secondary shear wave velocities (*V*p, *V*s), as shown in Table 6. A previous study also showed that an accelerated carbonation system enhanced the interface between the fibers and cementitious matrix in a sample, which improved the strong mechanical anchorage and interlocking effects [37] by filling the pores with calcite and fiber of the system. However, the findings of this study could play a significant role in improving the engineering properties of the soil using the MICP fiber matrix treatment.

## 4. Conclusions

This study was conducted to investigate the effects of the addition jute fibers to biocemented sand using MICP method. In this study, for the sand treatment using MICP method, the jute fiber contents were 0, 0.5, 1.5, 3, 5, 10, and 20% by sand weight and the lengths were 5, 15, and 25 mm. Based on the results of this study, the following conclusions could be outlined:

- Jute fiber has significant effects on the microbial performance, CaCO_3_ precipitation pattern, and solidification of sand. Using fluorescence microscopy, the survival capacity of the microorganisms was well demonstrated to be increased by the addition of jute fiber. The addition effectively improved not only the bacterial performance, but also the mechanical characteristics (UCS and ductility) of sand. The UCS of the sample increased with increasing fiber content; however, higher fiber addition past a point was found to decrease the UCS. From the results obtained in this study, the optimum jute fiber content was 3% and the optimum length was 15 mm;
- The CaCO_3_ precipitation was positively correlated with the addition of jute fibers, which yielded significant improvement of the engineering properties of the soil. The SEM analysis suggested that the added jute fiber coupled well with CaCO_3_ (i.e., CaCO_3_ was attached on and along the surfaces of fibers), forming reliable bridges within the soil matrix, which tended to limit the development of failure planes within specimens. This process potentially increased the strength and toughness of the treated specimens compared to those of control biocemented specimens (without jute fibers);
- As the amount and length of jute fibers increased beyond the optimum level, the fibers tended to become entangled with each other during preparation of the samples, which hindered the entry of bacteria and reduced the space available for bacterial survival and CaCO_3_ formation;
- In this study, natural jute fibers were used; however, the effects of chemically treated jute fibers and the roughness of jute fibers (surface roughness) have not been investigated in detail. In order to better understand the effects of fibers on soil stabilization (considering chemical pretreatment of the fiber, fiber roughness, etc.) using the MICP process, further studies are highly recommended.

## Figures and Tables

**Table 1:** The key features of the jute fibers used in this study.

Fiber Type	Thickness	Length (Total)	Weight	Type	Moisture Content	Colour
Jute	2 mm	510 m	900 g	Roll	3.4%	Golden-brown

**Table 2:** Testing conditions for CaCO_3_ precipitation with jute fibers (content).

Fiber Content [(%) mm]	CaCl_2_ (M)	Urea (M)	Bacterial OD_600_	Incubation Time (h)	Incubation Temperature (°C)
0	0.5	0.5	2	48	30
[(0.5) 15]	0.5	0.5	2	48	30
[(1.5) 15]	0.5	0.5	2	48	30
[(3) 15]	0.5	0.5	2	48	30
[(5) 15]	0.5	0.5	2	48	30
[(10) 15]	0.5	0.5	2	48	30
[(20) 15]	0.5	0.5	2	48	30

**Table 3:** Testing conditions for CaCO_3_ precipitation with jute fibers (length).

Fiber Length [(mm) %]	CaCl_2_ (M)	Urea (M)	Bacterial OD_600_	Incubation Time (h)	Incubation Temperature (°C)
0	0.5	0.5	2	48	30
[(5) 3]	0.5	0.5	2	48	30
[(15) 3]	0.5	0.5	2	48	30
[(25) 3]	0.5	0.5	2	48	30

**Table 4:** Testing conditions for the sand solidification test (syringe) considering fiber content (%).

Cases	Fiber Content [(%) mm]	Cementation Solution Injection	Bacterial Injection	Bacterial OD_600_	Curing Temperature (°C)	Curing Days
0	0	Everyday	Twice *	6	30	14
1	[(0.5) 15]	Everyday	Twice *	6	30	14
2	[(1.5) 15]	Everyday	Twice *	6	30	14
3	[(3) 15]	Everyday	Twice *	6	30	14
4	[(5) 15]	Everyday	Twice *	6	30	14
5	[(10) 15]	Everyday	Twice *	6	30	14
6	[(20) 15]	Everyday	Twice *	6	30	14

* Bacterial solution was injected at the beginning and after 7 days of the solidification test.

**Table 5:** Testing conditions for sand solidification test (syringe) considering fiber length.

Cases	Fiber Length [(mm) %]	Cementation Solution Injection	Bacterial Injection	Bacterial OD_600_	Curing Temperature (°C)	Curing Days
0	0	Everyday	Twice *	6	30	14
1	[(5) 3]	Everyday	Twice *	6	30	14
2	[(15) 3]	Everyday	Twice *	6	30	14
3	[(25) 3]	Everyday	Twice *	6	30	14

* Bacterial solution was injected at the beginning and after 7 days of the solidification test.

**Table 6:** Summary of test results for the biocemented sand after MICP treatment with jute fibers.

Fiber Content (%)	Unit Weight (g)	*V*s (km/s)	*V*p (km/s)	UCS (MPa)	Average CaCO_3_ (%)
0	65.6	0.92	1.12	0.5	2.4
0.5	64.2	0.87	1.22	1.5	9.3
1.5	63.5	0.95	1.24	1.4	11.88
3	60.1	0.92	1.25	1.6	13.29
5	61.5	0.9	1.23	1.3	7.9
10	60.2	0.99	1.22	0.8	4.6
20	59.9	0.93	1.24	0.3	3.29
**Fiber length (mm)**					
5	62.4	0.92	1.28	0.5	2.4
15	61.9	0.87	1.12	1.3	8.4
25	63.6	0.95	1.27	1.4	9.7
